# Long-term psychosocial response in Belgium after the 22/03/2016 terrorist attacks

**DOI:** 10.1093/eurpub/ckac129.335

**Published:** 2022-10-25

**Authors:** R Van Overmeire, L Vesentini, S Vanclooster, E Muysewinkel, J Bilsen

**Affiliations:** 1 Mental Health and Wellbeing Research Group, Vrije Universiteit Brussel, Brussels, Belgium; 2 Department of Public Health, Vrije Universiteit Brussel, Brussels, Belgium

## Abstract

**Introduction:**

While many studies have shown how terrorist attacks can have a mental health impact, there currently is little insight into how governments try to aid victims of terrorist attacks. In this study we viewed the long-term governmental psychosocial care response after the attacks of 22/03/2016 in Belgium, from both the level of the policy, as well as from the perspective of victims.

**Methods:**

This study employed a qualitative design. First, we studied guidelines, reports, policy documents and other relevant grey literature concerning the governmental psychosocial care response to the terrorist attacks. Second, we interviewed 27 victims of the terrorist attacks on their experiences with terrorist attacks for the micro level. We analyzed these interviews using a reflexive thematic analysis.

**Results:**

On the policy level, a problem occurred in the transfer from responsibilities from the federal level to the community's level. Furthermore, there was no proper psychotraumatology network of therapists, which did not allow for the proper therapy to be easily sought by victims. This was noticed also by the victims, who had issues finding a therapist that fitted their needs and could help with their trauma-related disorders. Furthermore, on both policy level and victim level, a reoccurring problem is the lack of recognition and knowledge of the mental health issues occurring after disasters such as terrorist attacks.

**Conclusions:**

The Belgian long-term psychosocial care response to the terrorist attacks of 22/03/2016 was not well organized and left some victims without proper aid. To improve the psychosocial response after terrorist attacks in the long-term, a combination of both more knowledge and recognition is needed.

